# Modeling Adipokine and Insulin‐Mediated Crosstalk Between Adipocytes and Beta Cells Using Flow‐Enabled Microfluidics

**DOI:** 10.1002/smll.202504686

**Published:** 2025-07-31

**Authors:** Mohamad Orabi, Mehdi Sh Yeganeh, Tae‐Hwa Chun, Joe Fujiou Lo

**Affiliations:** ^1^ Department of Mechanical Engineering University of Michigan Dearborn 4901 Evergreen Road Dearborn MI 48128 USA; ^2^ Department of Internal Medicine University of Michigan Ann Arbor MI 48109 USA; ^3^ Department of Internal Medicine Division of Metabolism Endocrinology and Diabetes Biointerfaces Institute University of Michigan Ann Arbor 22800 Plymouth Rd Ann Arbor MI 48109 USA

**Keywords:** 3T3‐L1, adipocytes, co‐culture, diabetes, INS‐1 beta cells, microfluidics

## Abstract

Obesity‐associated beta cell dysfunction is a crucial factor in the pathogenesis of Type 2 Diabetes (T2D), driven by a dysfunctional crosstalk between adipose tissues and pancreatic beta cells. Traditional culture systems cannot capture this crosstalk in a dynamic and controlled manner. A flow‐enabled microfluidic is developed with an embedded micro‐Tesla (µTesla) pump to assess the adipocyte‐beta cell crosstalk. This recirculating system allows them to study the transport of soluble‐factor‐based between cultured 3T3 L1 adipocytes and INS1 beta cells. It is found that flow‐enabled incubation with elevated glucose and insulin increased the levels of adipocyte‐derived secretions of IL‐6, TNF‐α, and adiponectin in the media. In turn, adipocyte‐derived IL‐6 enhanced beta‐cell insulin secretions in the same media, establishing a feed‐forward loop. This mechanism can contribute to the hyperinsulinemia and pro‐inflammatory conditions characteristic of obesity‐related T2D. The findings highlight the advantages of flow‐enabled microfluidics in modeling adipocyte‐beta cell crosstalk in obesity, providing novel insights into obesity‐associated beta cell dysfunction.

## Graphical abstract

A microfluidic system with a µTesla pump is developed to model adipocyte–beta cell crosstalk under flow. Elevated glucose and insulin enhanced adipocyte secretion of IL‐6, TNF‐α, and adiponectin, while IL‐6 stimulated beta‐cell insulin secretion. This feed‐forward loop mimics obesity‐driven beta cell dysfunction, offering new insights into Type 2 Diabetes pathogenesis.

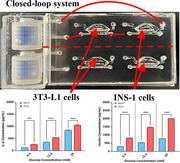

## 1 Introduction

Obesity is a significant public health challenge driven by a complex interplay of genetic, environmental, and lifestyle factors. The condition has attained epidemic proportions and impacts individuals across diverse ages, ethnicities, and socioeconomic backgrounds.^[^ ^1^, ^2^, ^3^, ^4^ ^]^ More importantly, obesity is the primary risk factor for developing insulin resistance and Type 2 diabetes (T2D).^[^ ^5^, ^6^ ^]^ Insulin resistance manifests as diminished insulin response, causing inadequate glucose uptake, unsuppressed lipolysis, and impaired glycogenesis.^[^ ^7^, ^8^ ^]^ In obesity, pancreatic beta cells increase insulin secretion, resulting in chronic hyperinsulinemia, further promoting adipose tissue expansion and inflammation. Furthermore, hyperinsulinemia with hyperglycemia negatively impacts adipose tissue function, increasing adipose‐derived inflammatory cytokines and potentially contributing to beta cell dysfunction.^[^ ^9^ ^]^ However, whether insulin resistance precedes beta cell dysfunction^[^ ^10^ ^]^ or chronic hyperinsulinemia leads to peripheral insulin resistance^[^ ^11^ ^]^ remains unclear. Therefore, developing an in vitro system to analyze adipocyte‐beta cell crosstalk could provide valuable insights into the physiological and pathological interactions between these metabolically active cells.

Adipose tissue inflammation in obesity negatively impacts insulin signaling.^[^ ^12^, ^13^, ^14^ ^]^ Adipose tissue produces inflammatory cytokines, like interleukin‐6 (IL6), interleukin‐1β (IL1B), and tumor necrosis factor‐alpha (TNF) in addition to metabolically beneficial adipokines, i.e., leptin (LEP) and adiponectin (ADIPOQ).^[^ ^15^ ^]^ Several molecular mechanisms may underlie the impaired insulin signaling in the insulin resistance state: TNF‐α induces resistance in adipose tissue by disrupting IRS‐mediated signaling and decreasing IRS1 and GLUT4 (SLC2A4) expression,^[^ ^16^ ^]^ TNF activates NF‐κB and JNK pathways, interfering with insulin receptor phosphorylation,^[^ ^17^ ^]^ IL‐6 may affect the secretion of adiponectin and leptin, which regulate insulin sensitivity, thereby contributing to insulin resistance.^[^ ^18^ ^]^

In rodents, glucose enters beta cells via Glut2 (Slc2a2) and is phosphorylated to glucose 6‐phosphate (G6P) by glucokinase (Gck), leading to ATP‐driven depolarization and calcium‐triggered insulin exocytosis.^[^ ^19^ ^]^ IL‐6 modulates beta cell function and induces protective autophagy and antioxidant responses, reducing oxidative stress and promoting beta cell survival under diabetogenic conditions.^[^ ^20^, ^21^ ^]^ However, chronic exposure to elevated IL‐6 may contribute to inflammation and beta‐cell dysfunction, potentially impairing insulin secretion.^[^ ^22^, ^23^ ^]^ TNF‐α negatively impacts beta cells by inducing inflammation, increasing oxidative stress, and promoting apoptosis.^[^ ^24^ ^]^ Our study focused on IL‐6, TNF‐α, and adiponectin as candidate adipokines mediating adipocyte‐beta crosstalk. Elucidating the regulation of these adipokines and their effects on insulin secretion helps us understand the mechanisms underlying adipocyte‐beta cell crosstalk.

In this study, we hypothesized that adipokines play a regulatory role in beta cell function while insulin modulates adipokine secretion. To test this hypothesis, we examined the molecular crosstalk between adipocytes and pancreatic beta cells using a micro‐Tesla (µTesla) pump‐driven recirculating microfluidics that allowed the medium to flow between the adipocytes and pancreatic beta cell culture compartments. Precise flow and control over experimental parameters are key advantages that make microfluidics well‐suited to model beta cell micro‐physiology and disease modeling.^[^ ^25^, ^26^ ^]^ Traditional flow sources such as syringes and pressure‐driven pumps have been widely used for microfluidic flow control.^[^ ^27^ ^]^ However, new integrated microfluidic pumps have emerged, such as acoustic, electroosmotic, electrolytic, and capillary siphoning‐based pumps.^[^ ^28^ ^]^ Despite innovations, many pumps fail to achieve steady‐state continuous recirculating laminar flows on microfluidic chips.^[^ ^29^ ^]^ On the other hand, the Tesla turbine can be used as a microfluidic pump, i.e., µTesla pump.^[^ ^26^ ^]^ Our novel application of µTesla pumps leverages boundary layer flows from the turbine to convert mechanical rotation into fluidic pressure, providing a constant, recirculating flow over cells cultured in microfluidic compartments.^[^ ^30^, ^31^ ^]^ Compared to traditional Petri dish or transwell techniques, our flow‐based coculturing provides more rapid and uniform cytokine transport in a circulatory manner that mimics the adipose‐beta cell communication in vivo. Using this µTesla microfluidics, we demonstrated that the adipocyte‐beta cell crosstalk synergistically enhanced glucose‐dependent insulin secretion and insulin‐driven adipokine release, suggesting a tightly coupled feed‐forward mechanism between adipocytes and pancreatic beta cells.

## 2 Experimental Section

### 2.1 Fabrication of a Flow‐Enabled Microfluidic Device Utilizing µTesla Pumps

**Figure 1:**
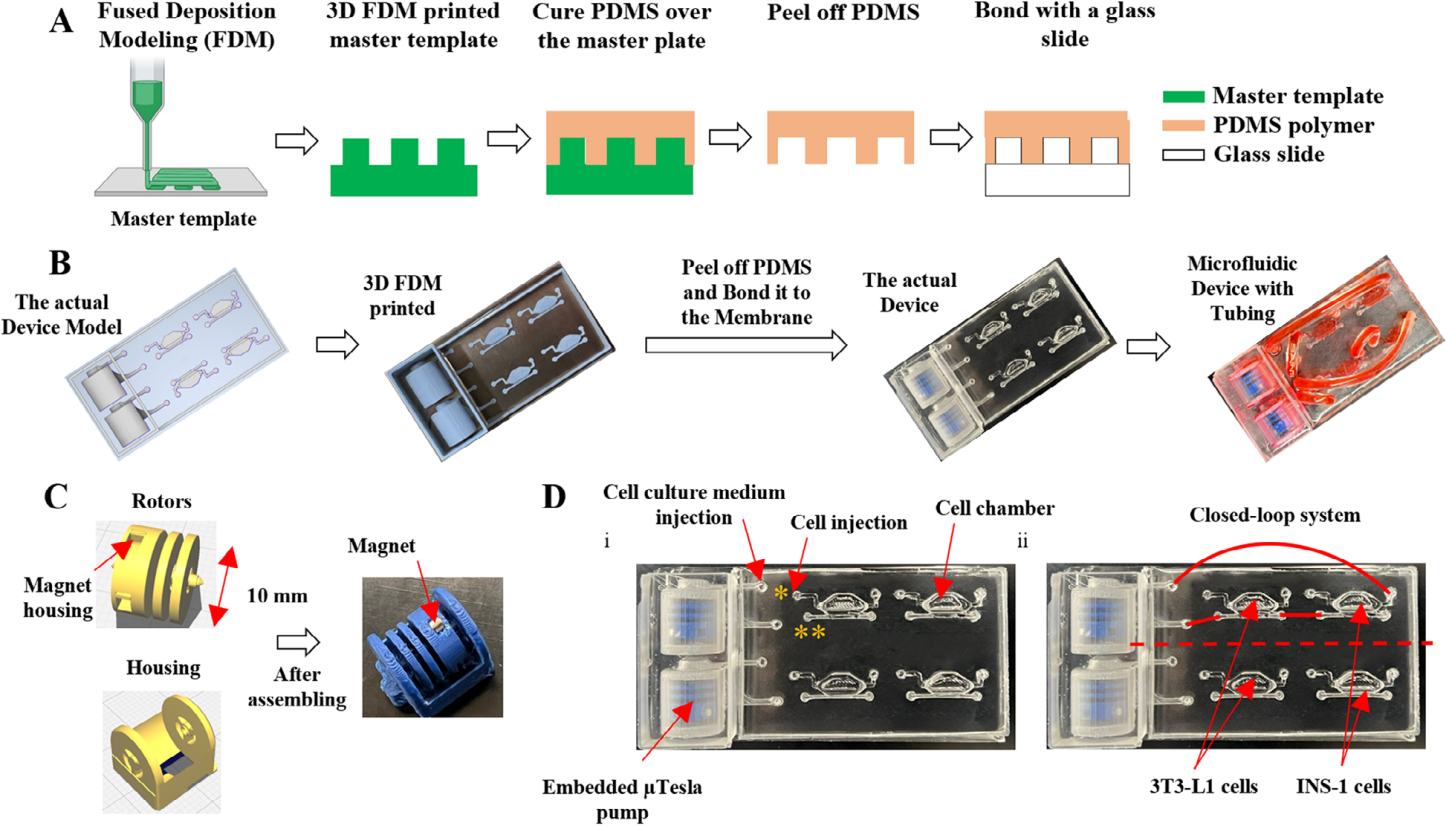
Fabrication of a Flow‐Enabled Microfluidic Device Utilizing µTesla Pumps A) The device was fabricated using Fused Deposition Modeling (FDM) to print the master template, over which polydimethylsiloxane (PDMS) was poured and cured. Once cured, the PDMS layer was peeled off and bonded to a glass substrate. B) The device design was created using Autodesk Inventor software and manufactured using the FDM method. µTesla pumps were integrated into the device, and the PDMS layer and the pumps were bonded to a glass substrate. C) µTesla pump comprises a rotor and housing of 10 mm size and is printed using a 0.15 mm nozzle following the FDM approach. Two magnets were polarized into the rotor before being assembled into the housing. D) These images illustrate injecting cell culture medium into the embedded µTesla pumps, connected via silicone tubes to the cell chambers, enabling media flow over the cultured cells. Cells were introduced into the chamber through the designated pore (^*^) and incubated overnight to facilitate attachment. Subsequently, the µTesla pump was connected to the cell chamber through a secondary pore (^**^), establishing a continuous media flow over the cells. The symmetrical design of the device allows us to run two experiments simultaneously with closed‐loop flow, providing a platform to study the bidirectional crosstalk between adipocytes and beta cells.

The microfluidic and µTesla components were designed using Autodesk Inventor Professional 2023 (AutoDesk. Inc, San Rafael, CA, USA) and printed via fused deposition modeling (FDM) on a custom‐built Voron Trident 3D printer with PolyLite ASA filament (Polymaker, Houston, TX, USA) (**Figure** **1**A,B).^[^ ^32^ ^]^ The µTesla pump rotor and the pump housing were printed at 240 °C using ASA filament with a 0.15 mm nozzle (Figure 1C; Figure S1, Supporting Information). The rotor is 1 cm, and the housing was designed to hold the rotor and allow it to rotate inside the housing. After printing, two magnets were inserted in the rotor in a polarized fashion. The rotor was inserted into the printed pump housing to assemble the µTesla pump (Figure 1B). The fabricated µTesla pump was soaked in 1 m hydrochloric acid (HCL) to increase positive surface charges and then washed with distilled water 5 times. After the HCL incubation, the pump was incubated in 1% sodium dodecyl sulfate (SDS) for 2 days for sterilization and washed with PBS before device encapsulation. For all experiments, the µTesla pump integrated microfluidic device was operated on a mini stir plate after sterilization.

The microfluidic channels were also printed in ASA at 240 °C using a 0.15 mm nozzle. A wall surrounding the microchannels was printed to allow PDMS molding (Figure 1A,B). While the device was printing, PDMS prepolymer was prepared and degassed to save time. It was poured onto the 3D‐printed mold directly on the print bed and baked at 90 °C for 2 h. The cured PDMS was then peeled from the print bed and bonded with a glass slide encapsulating a pre‐printed µTesla pump using plasma bonding. The bonded device was baked at 100 °C on a hot plate for 2 h before device sterilization for cell culturing. The device has 2 cell chambers on each side, which allows the co‐culture of 3T3‐L1 adipocytes with INS‐1 rat pancreatic beta cells in a closed‐loop manner (Figure 1D). Under flow conditions, media was introduced into the µTesla pump and operated for ≈5 min to release air bubbles while adding the media. Monoculture studies were performed under static and flow conditions, however, this device was designed for co‐culture experiments under flow conditions. The total volume of media, including the pump, cell culture chambers, and tubing, is 1 mL.

### 2.2 COMSOL Molecular Diffusion Modeling

To illustrate the critical role of recirculating microfluidics in modeling the complex pathophysiology of diabetes with in situ bio‐detection, COMSOL Multiphysics finite element modeling was employed to simulate molecular diffusion on the surfaces of the microfluidic device in comparison to a Transwell plate. Laminar Flow and Transport of Dilute Species were coupled using COMSOL software and simulated in a time‐dependent analysis over a 500 s duration. The flow rate was set to 950 µL min^−1^, matching the experimental condition. Fluid density of 1020 kg m^−1^ with a dynamic viscosity of 1 cP was used to approximate the cell culture media. A diffusion coefficient of 1.53 × 10⁻¹⁰ m^2^ s^−1^ was used to model insulin transport in the microfluidics.^[^ ^33^ ^]^ Insulin, with a smaller diffusion coefficient than IL6, was assumed to provide a lower bound limit to the transport for the modeling of both molecules.

### 2.3 Adipocyte Differentiation and Experimental Setup

**Figure 2:**
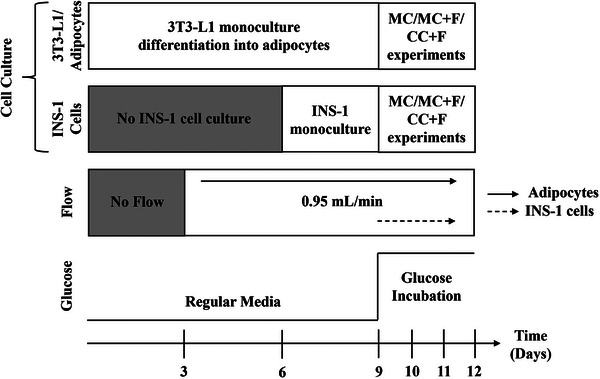
Timeline for the adipocyte differentiation and experimental setup 3T3‐L1 preadipocytes were differentiated into adipocytes in a microfluidic chamber. On the 6th day of the 3T3‐L1 differentiation, beta INS‐1 cells were plated in another chamber for 3 days until they became confluent. The flow of media was started on the 3rd day of 3T3‐L1 adipocyte differentiation for adipocyte monoculture with flow (MC+F) and on the 9th day for INS‐1 cell MC+F and the co‐culture of 3T3‐L1 adipocytes and INS‐1 beta cell with flow (CC+F). For the monoculture condition (MC), no flow was applied (static condition).

3T3‐L1 preadipocytes (CL‐173, ATCC) were cultured in high glucose (4.5 g L^−1^ glucose) with L‐Glutamine Dulbecco's modified Eagle's medium (DMEM) supplemented with 10% (v/v) bovine calf serum (BCS) and 1% (v/v) penicillin‐streptomycin (regular media). 1 × 10^6^ 3T3‐L1 cells were loaded onto the device in 1 mL of the regular medium and cultured in a CO_2_ incubator at 37 °C for 3 days until the cells reached 70% confluency. Adipogenesis was induced with adipogenic media comprising regular media, 10 nM triiodothyronine (T3),10 µm troglitazone, 0.25 µm dexamethasone, and 1 µg mL^−1^ insulin, and the devices were incubated for an additional 3 days. Adipogenic media was replaced with 3T3‐L1 regular media supplemented with 1 µg mL^−1^ insulin, and the cells were cultured for an additional 3 days to complete adipogenesis (**Figure** **2**). Beta INS‐1 cells (C0018007, AddexBio) were cultured in the device on the 6th day of the differentiation process and left for 3 days to reach 90% confluency before co‐culturing with adipocytes (Figure 2). INS‐1 beta cells medium is composed of RPMI 1640 medium, no glucose supplemented with 2 mM L‐Glutamine solution, 1 mm sodium pyruvate, 10 mm HEPES, 50 µm 2‐Mercaptoethanol, 10% (v/v) fetal bovine serum (FBS), and 1% (v/v) penicillin‐streptomycin. All cell images were taken with an Olympus IX75 microscope using 10×, 20×, and 50× objective lenses.

High‐glucose DMEM (25 mm) was diluted with no‐glucose DMEM to prepare 5.5, 12.5, and 25 mm glucose DMEM for adipocyte monoculture, maintaining L‐glutamine (4 mm), sodium pyruvate (1 mm), and sodium bicarbonate (1500 mg L^−1^). For INS‐1 culture, glucose was directly added to no‐glucose RPMI 1640 to achieve the same concentrations. Co‐culture media were prepared by mixing adipocyte and beta INS‐1 media at 1:1 ratio. Cells remained immobile in the device as they were cultured in 2D within the chambers. They were not exposed to flow during the culturing period. However, after activating the pump, the media was changed every 8 h to clear debris and maintain pH stability.

### 2.4 Cell Viability Assays

Cell viability was determined with a Live/Dead Cell Imaging Kit. After cell attachment to the device, they were washed with PBS and incubated with calcein‐AM (green fluorescent) to indicate intracellular esterase activity, followed by ethidium homodimer‐1 to indicate loss of plasma membrane integrity. After 30 min of incubation, the device was put in an on‐stage incubator under an Olympus IX75 microscope for 72 h. Images were taken via the automated X/Y stage to generate image collages at 24, 48, and 72 h (Days 10, 11, and 12). ImageJ software was then used to quantify and analyze the images. All measurements were conducted in triplicate, and cell viability was calculated.^[^ ^34^ ^]^

### 2.5 Metabolic Activity of Cells

On days 9, 10, 11, and 12, activated XTT reagent (100 µL of 10 µm tetrazolium dye, XTT reagent, and 2 µL of 1 µm activation reagent (ATCC, Manassas, VA)) was added to the cells along with fresh media and incubated at 37 °C and 5% CO_2_ for 4 h. Samples were collected. The absorbance was then measured using a BioTek Eon Microplate reader at a wavelength of 450 nm. The metabolic activities were calculated as normalized absorbance on day 9. The data were collected from at least three independent experiments, each carried out in three replicates.

### 2.6 Differentiation Percentage and Lipid Droplet Size

Image J was used to assess the percentage of differentiation and the size of lipid droplets. For differentiation analysis, cells containing lipid droplets were categorized as differentiated, while those lacking droplets were considered undifferentiated. The differentiation percentage was determined by dividing the number of lipid‐containing cells (classified as particles in Image J) by the total number of cells. Additionally, Image J was utilized to measure the size of lipid droplets within adipocytes. Oil‐Red‐O staining is a commonly used experimental technique to detect adipocyte lipid content. Cells were washed once in PBS and fixed using the provided fixative solution at room temperature for 15 min. The fixative solution was removed and washed 3 times with deionized water before adding the Oil Red O working solution. Samples were then incubated for 15 min at room temperature and washed five times with deionized water. Images were captured using an Olympus IX75 microscope with a 50× magnification lens, and the droplet size was analyzed and quantified using ImageJ.

### 2.7 Mitochondrial ATP Quantification

BioTracker ATP‐LW Live Cell Dye was used to measure mitochondrial ATP production in 3T3‐L1 adipocytes and beta INS‐1 cells. For adipocytes, after 12 days of incubation with a differential medium in the microfluidic device, the medium was removed and replaced with 1 mL regular medium containing 5 µm BioTracker dye. For 3T3L1 and beta INS‐1 cells, after 3 days of co‐culture, samples were stained with 5 µm BioTracker Dye. All the samples were incubated for 30 min at 37 °C and 5% CO_2_ before moving them to the microscope for imaging. Samples were incubated for 24 h, and images were captured using an Olympus IX75 microscope with a 10× objective lens at 0, 3, 12, and 24 h. Fluorescence signal intensity was quantified using ImageJ and normalized to the level at hour 0.

### 2.8 Reactive Oxygen Species Quantification

Reactive oxygen species (ROS) were qualitatively detected using the cell‐permeant 2’,7’‐dichlorodihydrofluorescein diacetate (H_2_DCFDA). H_2_DCFDA is a chemically reduced form of fluorescein to detect reactive oxygen intermediates in neutrophils and macrophages. Upon cleavage of the acetate groups by intracellular esterases and oxidation, H_2_DCFDA converts from a non‐fluorescent state to a highly fluorescent 2’,7’‐dichlorofluorescein (DCF), indicating the presence of ROS in cells. Per the manufacturer's instructions, the DCFDA was dissolved in DMSO for a 10 mm standard solution. The solution was then diluted 2 times to get a concentration of 10 µm. 1.2 µL of 10 µm solution was added to the regular media and incubated for 1 h. Samples were incubated in the media for 24 h, and images were captured using an Olympus IX75 microscope with a 10x objective lens at 0, 3, 12, and 24 h. Fluorescence signal intensity was analyzed and quantified using ImageJ and normalized to the signal at hour 0.

### 2.9 Adipokine and Insulin Quantification

IL‐6 concentrations were measured using an IL‐6 Mouse ELISA Kit. Samples were collected 24 h after adding 50, 100, 300, 1000, and 10000 pg mL^−1^ of insulin to the flowing media, and the absorbance was then measured using a BioTek Eon Microplate reader at 450 nm. TNF‐α, insulin, and adiponectin secretion were quantified following the protocol described by TNF‐α mouse, insulin rat, and mouse adiponectin ELISA kits, and the absorbance was measured the same way as IL‐6 at 450 nm.

### 2.10 Calcium Influx Detection

For calcium responses with IL‐6 modulations, INS‐1 cells were stained with cell‐permeant 5 µm Fura‐2 AM for 30 min, then the dye was replaced with glucose‐free Krebs‐Ringer buffer for 5 min. The devices were then placed under fluorescence microscopy and incubated for 5 min for calibration. Cells were stimulated with 12.5 mm glucose for 30 min with different IL‐6 concentrations. FURA fluorescence intensities were measured at 340 and 380 nm wavelengths at the 24th h, and the ratio of 340/380 nm intensity was calculated to represent intracellular calcium levels.

For calcium responses with glucose incubations, all cells were incubated at their respective concentrations of 5.5, 12.5, and 25 mm for their specific durations. Post‐incubation, the cells were stained with FURA‐2 AM for 30 min and then with Krebs‐Ringer buffer without glucose for 5 min. Afterward, the dye was replaced with buffers containing the 5.5, 12.5, and 25‐mm glucose concentrations corresponding to their incubation conditions for another 30 min to assay their calcium responses. Measurements were then conducted in the same way described above.

### 2.11 Statistical Analysis

All data were presented as means ± SEM. Figures were generated using BioRender, and data was statistically analyzed using Prism – GraphPad. Outliers were identified, normality was tested, and statistical significance was assessed accordingly. A one‐way ANOVA was applied for normally distributed data, and a two‐way ANOVA was used when two independent variables were involved. A nonparametric one‐way ANOVA was used when the data deviated from a normal distribution.

## 3 Results

To examine the effect of flow on metabolic cell function, 3T3‐L1 adipocytes and INS‐1 beta cells were cultured on‐chip as monoculture (MC) separately without and with flow (MC+F). Next, the two cell types were co‐cultured under flow conditions (CC+F) to determine the effects of co‐culturing adipocytes and beta cells compared to MC+F. Under the no‐flow condition, cells were statically cultured, and the media were manually changed every 8 h using a syringe pump to avoid acidity of the media due to the high metabolic activity of adipocytes. For the MC+F condition, chambers containing adipocytes or beta INS‐1 cells were individually connected to the µTesla pump, and media were changed every 8 h. For co‐culture studies, the same media is recirculated through both chambers (adipocytes and beta cells) in a closed‐loop system, with the media changed every 8 h (Figure 1D).

### 3.1 Advantages of µTesla Pump‐Equipped Microfluidics

**Figure 3:**
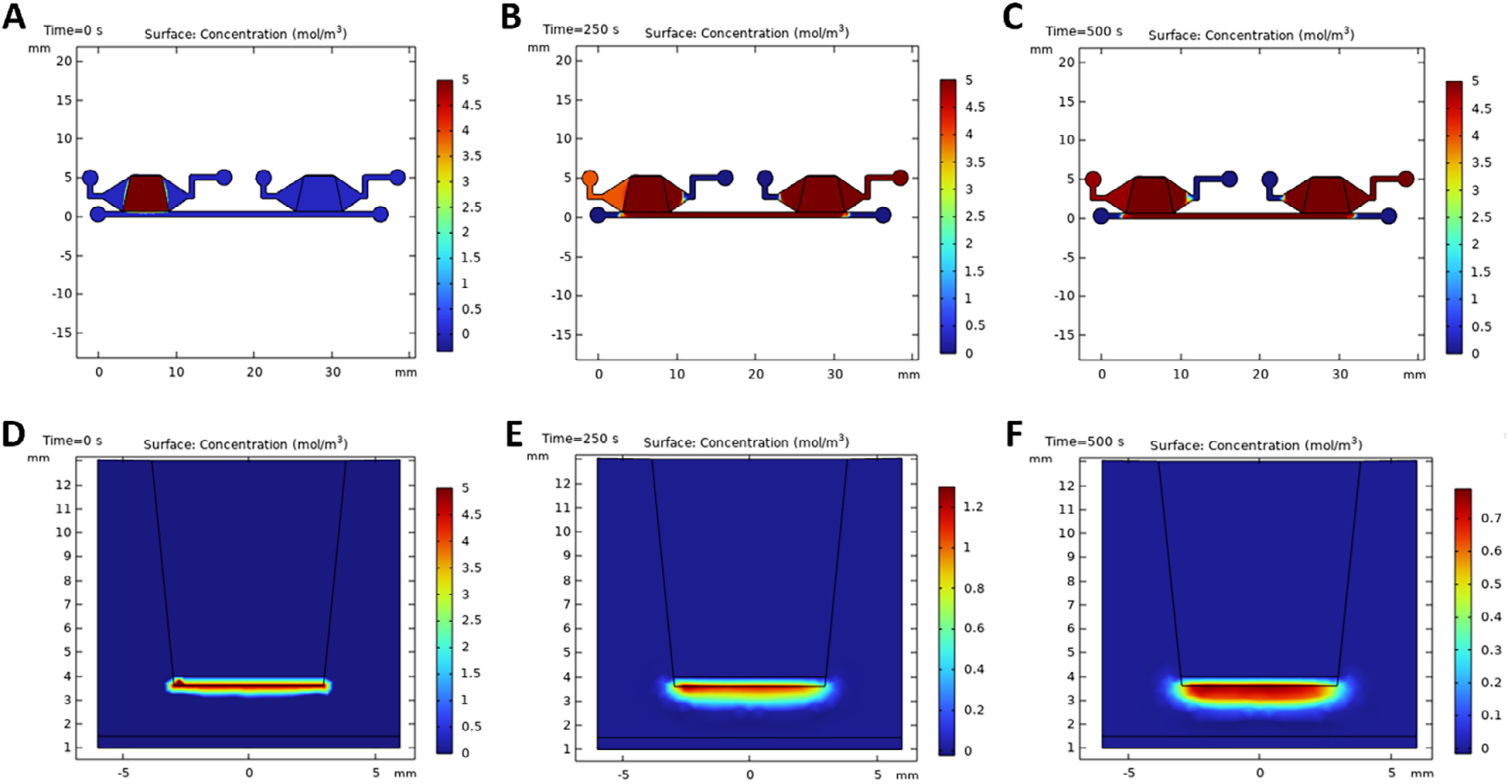
Molecular diffusion modeling in Transwells versus a closed‐loop microfluidic device. In the microfluidic setup, adipocytes are placed in the left chamber and beta cells in the right, with left‐to‐right recirculating flow at 950 µL min^−1^ shown at 0 s A), 250 s B), and 500 s C). The simulation accounts for the delay time associated with fluid recirculation through the pump system. For comparison, the Transwell configuration with adipocytes in the insert and beta cells at the bottom is shown at corresponding time points: 0 s D), 250 s E), and 500 s F).

Microfluidic techniques provide several advantages for disease modeling and in vitro experiments, including precise control of microenvironments, high sensitivity, and faster kinetics.^[^ ^35^ ^]^ A common hesitation in using novel microfluidics is that they only augment but still require existing biomolecular techniques for subsequent validation. However, diabetes involves multiple tissues, circulating cells, and signaling molecules. Standard methods cannot provide quantitative control or detection involving perfusates and different cell types. Thus, recirculating microfluidics with in situ bio‐detection is key in modeling the complex disease of diabetes.^[^ ^36^ ^]^ In addition to replicating multi‐cellular and physiological circulation, recirculating microfluidics can amplify cell‐cell signaling due to the microliter circulation volumes, translating to better detection sensitivity and enhanced cell responses. The conventional transwell co‐culture model requires a large media volume and relies on passive diffusion, leading to slow and non‐directional delivery of cell‐derived factors. In contrast, a microfluidic device enables rapid ligand delivery to target cells, achieving a steady state. Existing microfluidic pumps–rotor, peristaltic, and electroosmotic pumps – have limited pump volume, cause variable pulsatility, and cannot recirculate flows. Our proposed µTesla pump integration is a breakthrough in providing continuous, laminar flow over adipocyte and β‐cell cultures on a chip^[^ ^25^ ^]^ (**Figure** **3**). With precise flow down to the µL min^−1^ range, the µTesla can mimic the physiological flow rate required for pancreatic blood flow. Furthermore, our multi‐modal detection of IL6, insulin, and TNF‐α reduces variabilities and corroborates measurements. Using this innovative microfluidics, we aim to define adipocyte‐beta cell crosstalk as critical to the pathogenesis of obesity‐induced T2D.

Computational fluid dynamics (CFD) was conducted to demonstrate that the average wall shear stress within the chamber was ≈1.1792 mPa. This value is significantly lower than the commonly reported thresholds (0.1–0.3 Pa) that affect or damage adherent cells.^[^ ^37^ ^]^ Therefore, our design ensures a biocompatible mechanical microenvironment for sensitive cell types such as INS‐1 beta cells. To further illustrate these findings, Figure S11A (Supporting Information) presents a surface plot of the computed shear stress distribution within the chamber, confirming uniformly low stress levels across the entire culture region. Additionally, Figure S11B (Supporting Information) shows a line profile plot of shear stress along the central axis of the chamber, highlighting the gradual transitions in velocity and stress throughout the chamber length. These visualizations further support the conclusion that the microfluidic environment maintains cell‐friendly shear conditions.

First, the impact of the flow‐based microfluidic on viability was characterized via live‐dead assays over 48 h. Overall viabilities of adipocytes and beta cells were not different between monoculture (MC) versus monoculture with flow (MC+F) at 72.03% and 68.67% for adipocytes and 63.16% and 60.89% for beta cells, respectively (p = 0.24 for adipocytes and p = 0.13 for beta cells, day 12, Figure S3, Supporting Information). However, co‐culturing with flow (CC+F) improved viability of adipocyte and INS1 (p< 0.0001, for both on day 12) by reducing cell death. Absorbance‐based proliferation data corroborated this co‐cultured effect, Figures S4 and S5 (Supporting Information).

Furthermore, the effects of flow‐based microfluidics on 3T3‐L1 differentiation were also characterized. Media flow significantly improved adipocyte differentiation of 3T3‐L1 cells, with a higher percentage of lipid‐containing cells observed in MC+F than MC (60.4% vs 55.5%, p = 0.0009, Figure S6A,B, Supporting Information). Co‐culturing with beta INS‐1 cells (CC+F) showed increased lipid accumulation relative to MC but not MC+F (p< 0.0001 vs p = 0.08, Figure S6A, Supporting Information). Additionally, flow was associated with increased lipid droplet size: 7.9 ± 0.6 µm without flow (MC) versus 10.1 ± 0.8 µm with flow (MC+F) (p = 0.019), which was further increased under co‐culturing: 15.5 ± 0.9 µm in CC+F (p = 0.0015, MC+F vs CC+F) (Figure S6B, Supporting Information).

**Figure 4:**
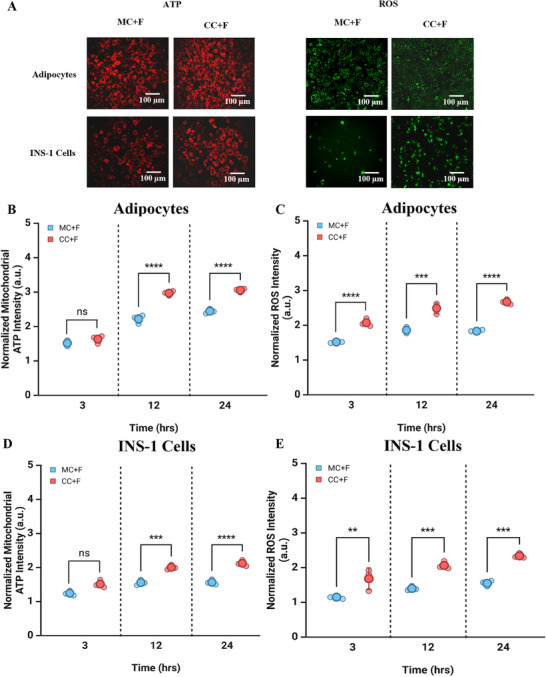
Co‐culture with flow improves the metabolic activities of adipocytes and beta cells A) Cellular ATP (red) and ROS (green) productions in adipocytes (top) and INS‐1 beta cells (bottom). Images were taken for 24 h post the 3 days of INS‐1 culture and post‐differentiation (9 days) for adipocytes, and then quantified using image J. B) Quantified ATP production in adipocytes. n = 3 independent experiments. C) Quantified ROS production in adipocytes. n = 3 independent experiments. D) Quantified ATP production in INS‐1 beta cells. n = 3 independent experiments. E) Quantified ROS production in INS‐1 beta cells. n = 3 independent experiments. Two‐way ANOVA with Tukey multiple comparisons test was used for statistical analyses. ^*^ *p*< 0.05, ^**^ *p*< 0.01, ^***^ *p*< 0.001, ^****^ *p*< 0.0001. n stands for different experiments conducted in separate days.

We also characterized the mitochondrial ATP and reactive oxygen species (ROS) of monocultured 3T3‐L1 cells with and without a flow (Figure S8, Supporting Information) and adipocytes and INS‐1 beta cells with flow in monocultured and cocultured conditions (**Figure** **4**). 3T3‐L1 cells exhibited higher mitochondrial ATP synthesis with flow (MC+F) compared to no flow (MC) (Figure S8, Supporting Information), reflecting their increased mitochondrial activities. Flow and co‐culturing increased adipocyte (Figure 4A,B) and, to a greater degree, beta cell mitochondrial ATP levels (Figure 4A–D). The trend for ROS followed that of mitochondrial ATP for both cell lines (Figure 4A,C,E).

### 3.2 Flow Augments the Response of Adipocytes and INS‐1 Beta Cells to Insulin and IL‐6

We hypothesized that adipokines play a significant role in regulating pancreatic beta cell function. We first looked at adipocytes MC to quantify their IL‐6, TNF, and adiponectin secretions, which showed a dose‐dependent response to insulin incubation (Figure S9A–C, Supporting Information). Notably, flow induction had a strong effect on TNF secretion, while its effect on adiponectin was smaller (p< 0.001 for all). On the beta cell side, IL‐6 incubation increased both calcium flux and insulin secretion (p< 0.0001 for both), Figure S9D,E (Supporting Information). Here, flow had a stronger effect on calcium response than insulin secretion, suggesting calcium‐independent mechanism is in play.^[^ ^38^ ^]^ These results together showed a potential synergy between adipocyte IL‐6 and beta cell insulin secretions when co‐cultured in a flow‐dependent manner, prompting us to study them in further detail.

### 3.3 Adipocyte‐Beta Cell Co‐Culture Under Flow Exhibits a Robust Response to Glucose

After observing the insulin‐dependent induction of IL‐6 from adipocytes and IL‐6‐dependent induction of insulin from beta cells, we hypothesized that insulin and IL‐6 may play a key role in glucose‐dependent metabolic crosstalk between adipocytes and beta cells.

**Figure 5:**
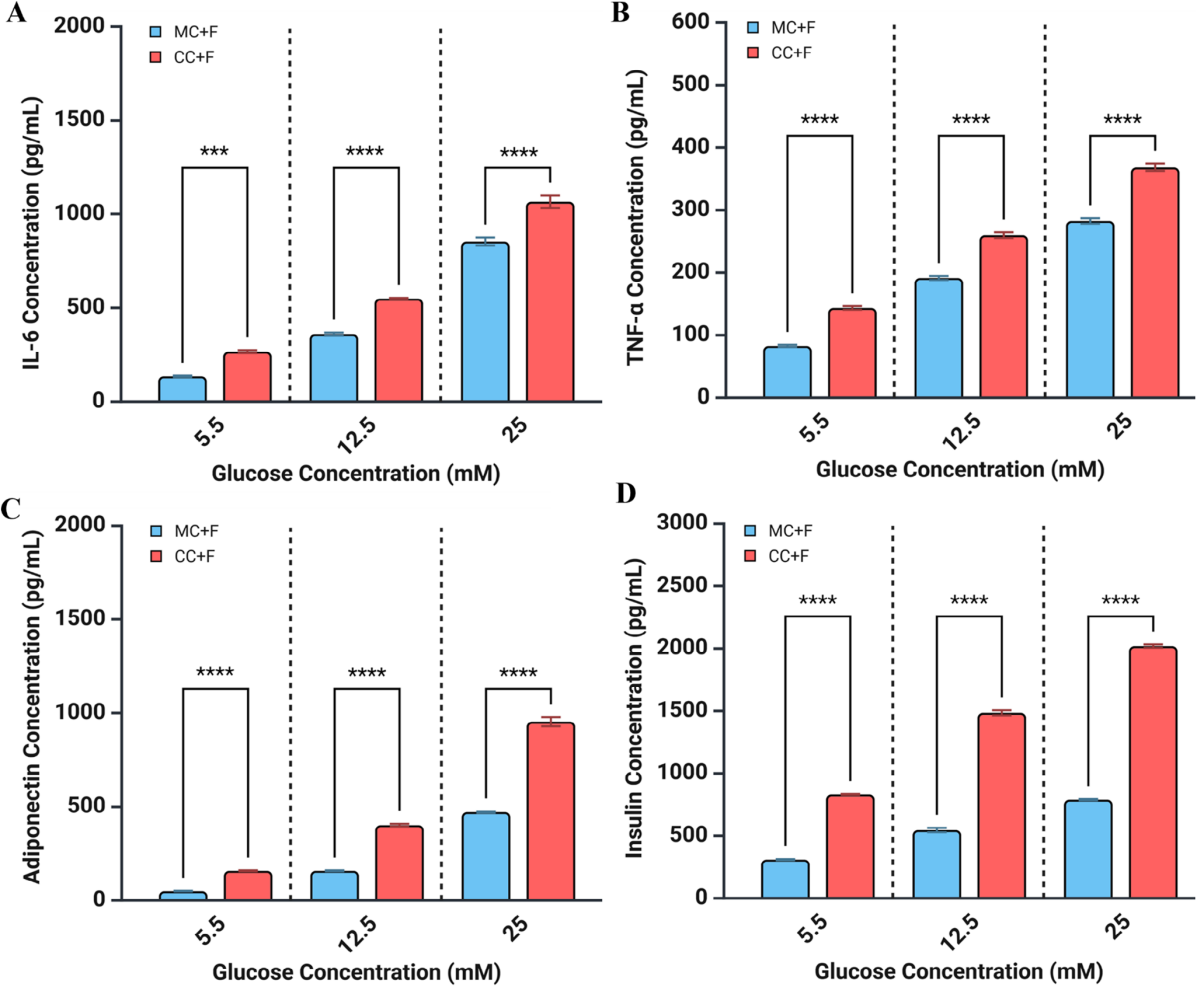
Adipocyte‐beta cell co‐culture under flow responds to hyperglycemia A) Adipocyte IL‐6 response to glucose incubation in MC+F and CC+F conditions. Adipocytes were incubated with different concentrations of glucose (5.5, 12.5, 25 mm) for 24 h under MC+F and co‐culture with beta cells with flow (CC+F) and IL‐6 levels in the media were measured. B) Adipocyte TNF‐α levels in adipocyte MC+F and adipocyte‐beta cell CC+F in response to increasing concentrations of glucose. C) Adiponectin levels in adipocyte MC+F and adipocyte‐beta cell CC+F in response to increasing glucose concentration. D) Insulin levels in INS‐1 cell MC+F and adipocyte‐beta cell CC+F in response to glucose. Two‐way ANOVA with Tukey multiple comparison tests was used. Data are presented as means ± SEM, n = 3 independent experiments. ^*^ *p* < 0.05, ^**^ *p* < 0.01, ^***^ *p* < 0.001, ^****^ *p* < 0.0001. n stands for different experiments conducted in separate days.

Subsequently, adipocytes and beta cells in MC+F and CC+F conditions were cultured in the media with glucose 5.5 (baseline), 12.5, and 25 mm to determine the effect of hyperglycemia over 24 h. IL‐6 secretion increased with higher glucose concentrations and was further enhanced by co‐culturing under flow (MC+F vs. CC+F) (**Figure** **5**A). Like IL‐6, TNF‐α showed a significant response to increased glucose concentrations plus enhancements under co‐culture conditions (Figure 5B). Increasing glucose dramatically enhanced adiponectin secretion, particularly under co‐culturing, exhibiting a strong exponential trend (Figure 5C). Hyperglycemia, especially under co‐culture conditions, significantly affected the secretion of IL‐6, TNF‐α, and adiponectin (p < 0.0001 for all), indicating its pervasiveness in adipokine regulation across these markers.

For INS1 cells, basal calcium influx under glucose incubation was enhanced under the synergistic effects of flow and co‐culture. Specifically, co‐culturing enhancements were more dramatic under hyperglycemia (12.5 and 25 mm) conditions (Figure 5D), with corresponding increases in insulin secretion. This suggests that the adipocyte‐beta crosstalk leads to greater hyperglycemia‐induced insulin secretion, compared to the modest response in monocultures (p < 0.0001).

### 3.4 Adipocyte‐Beta Cell Co‐Culture with Flow Elevates Hyperglycemic Responses Over 3 Days

**Figure 6:**
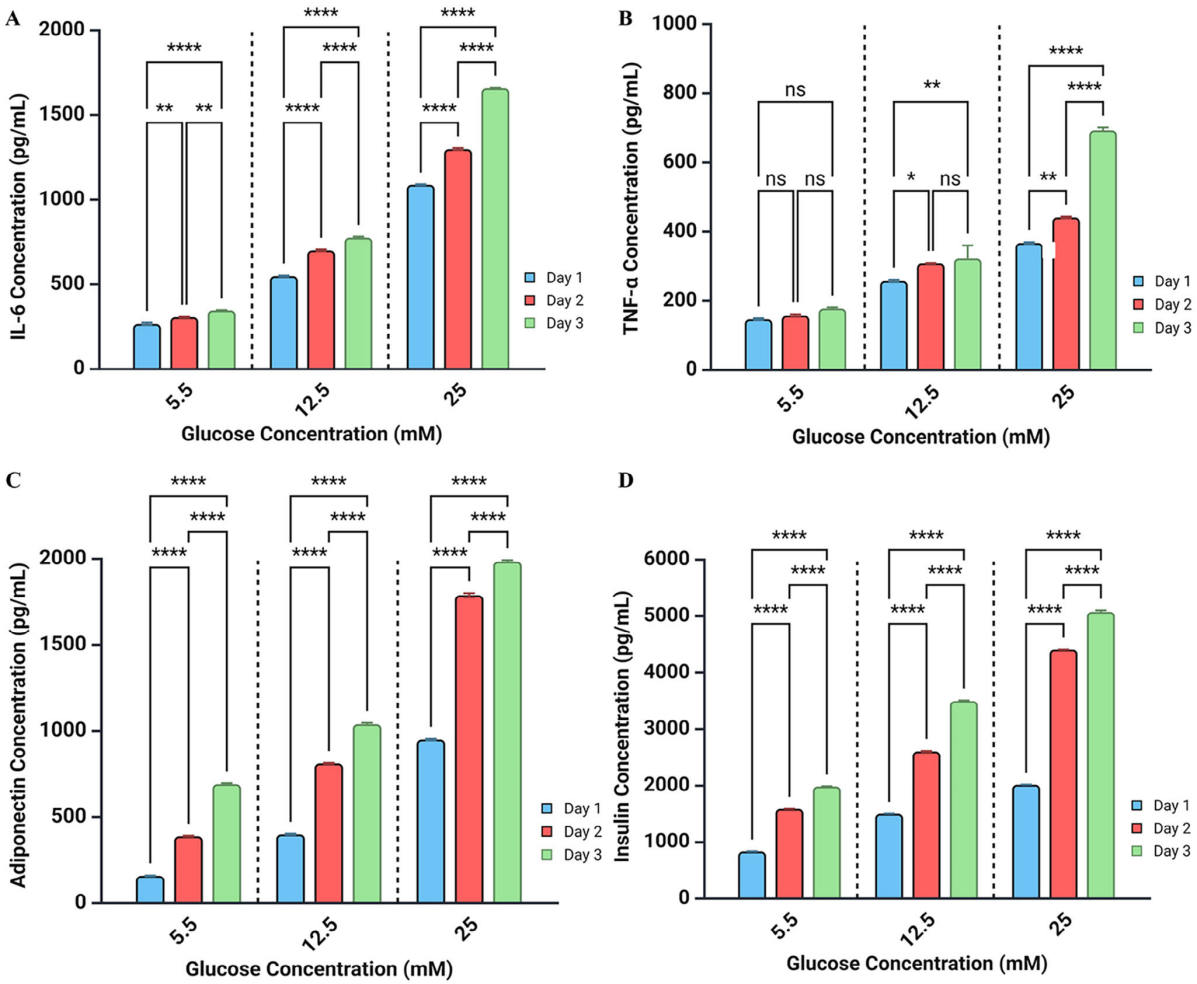
Adipocyte‐beta cell co‐culture with flow maintains a robust response to glucose for up to 3 days A) IL‐6 levels in adipocyte‐beta cell CC+F in response to different glucose concentrations. Measured on days 1, 2, and 3. B) TNF‐α levels in adipocyte‐beta cell CC+F in response to different glucose concentrations. C) Adiponectin levels in adipocyte‐beta cell CC+F in response to different glucose concentrations. D) Insulin levels in adipocyte‐beta cell CC+F cultured in the media with different glucose concentrations for up to day 3. Assays were performed with media collected every day, 24 h after daily medium change. After media collection, samples were frozen at −20 °C until used for ELISA assays. Two‐way ANOVA with Tukey multiple comparisons test was used for statistical analysis. Data is presented as means ± SEM, n = 3 independent experiments. ^*^ *p* < 0.05, ^**^ *p* < 0.01, ^***^ *p* < 0.001, ^****^ *p* < 0.0001, ns: no significance. n stands for different experiments conducted in separate days.

Extended co‐culturing under hyperglycemia conditions was evaluated for an additional two days, with culture media replaced every 24 h. Media IL‐6 and TNF levels increased with glucose concentration and over the 3 days of incubation. (**Figure** **6**A,B). Notably, 25 mm glucose elicited the most pronounced increases of IL‐6 and TNF at day 3 over days 1 and 2. On the other hand, adipocyte adiponectin and INS1 insulin levels saw greater responses over the 3 days of incubation (Figure 6C,D). This is especially poignant at 25 mM glucose, where levels more than doubled from day 1 to day 3, for both cell type and their respective secretions. Consistent with the earlier findings on Day 1, these results suggest that the adipocyte‐beta cell crosstalk model under extended high‐glucose conditions may replicate hyperglycemia‐induced hyperinsulinemia and a pro‐inflammatory state.

## 4 Discussion

In this study, we investigated the effects of flow‐based co‐culturing and hyperglycemia on the circulating levels of adipokines and insulin. In our supplementary results, applying flow to cells stimulated 3T3 lipid droplet accumulation, increased mitochondrial ATP and ROS expressions, and facilitated the reciprocal incubation between beta cells and adipocytes. Interestingly, tissue perfusion is reduced in obesity‐dependent T2D,^[^ ^39^ ^]^ potentially reducing these benefits of flow. Moreover, when subjected to hyperglycemia, the flow‐based co‐culture more than doubles the IL‐6 and insulin secretions compared to their monoculture levels and continued to rise under days of extended exposures. This suggests that the positive effects of flow and coculturing may become pathological if the balance is perturbed by significant, extended hyperglycemia. On the other hand, lipid droplet accumulation is part of adipogenesis, where adipocytes mature through a cascade of morphological, biomolecular, and gene expression changes. Lipid accumulation and fatty acid exposure can increase glucose‐stimulated insulin secretion through surface receptors on beta cells. Thus, chronic exposure to fatty acids combined with elevated glucose may further impair the viability and function of beta cells.^[^ ^40^ ^]^ Taken together, adipose flow response via adipogenesis and altered adipokine secretions may be instrumental in metabolic homeostasis, inflammation, and downstream insulin sensitivity.^[^ ^41^, ^42^ ^]^

Metabolic inflammation manifests in various tissues involved in nutrient regulation, with adipose tissue being a significant player in T2D.^[^ ^43^, ^44^ ^]^ The observation of elevated TNF‐α in the adipose tissue of obese individuals signifies a strong link between obesity, diabetes, and chronic inflammation.^[^ ^45^ ^]^ Proinflammatory adipokines, including TNF‐α and IL‐6, and anti‐inflammatory adipokines, including adiponectin, correlate with obesity‐related complications, fostering a persistent, low‐grade inflammatory state.^[^ ^46^ ^]^ In T2D, diminished insulin secretion from pancreatic beta cells, attributed to factors like insulin resistance or beta cell dysfunction, is common.^[^ ^47^ ^]^ INS‐1 cells serve as a model for reflecting this dysfunction in vitro, with calcium playing a critical role in insulin secretion.^[^ ^48^ ^]^ Glucose metabolism in beta cells increases intracellular calcium levels, leading to insulin exocytosis.^[^ ^49^ ^]^ However, dysregulation of calcium homeostasis in diabetes can impair insulin secretion.^[^ ^50^ ^]^ Our study unveiled an intriguing interaction between adipocytes and INS‐1 cells under flow with no shear (Figure S11, Supporting Information), where adipokines are augmented, and insulin secretion with calcium influx is elevated, especially under hyperglycemia. Consistent with the mitochondrial responses, extended hyperglycemia may disrupt the physiological crosstalk between INS‐1 and adipocytes with increased TNF‐α, IL‐6, and insulin levels, leading to aggravated inflammatory state and beta cell stress with excess calcium influx and insulin production.^[^ ^51^ ^]^ As beta cell function diminishes, insulin secretion becomes insufficient to counteract insulin resistance, leading to impaired glucose tolerance and eventually the onset of diabetes.^[^ ^52^, ^53^ ^]^ In our monocultures, IL‐6 incubation increased insulin secretion from INS‐1 cells. On the other hand, insulin‐stimulated adipocytes upregulated adipokine release. Extended hyperglycemia augmented TNF‐α secretion from adipocytes. TNF‐α can affect insulin receptor autophosphorylation, impairing insulin‐dependent glucose uptake.^[^ ^54^ ^]^ TNF‐α can also blunt adiponectin secretions, further affecting insulin resistance in distal tissues.^[^ ^55^ ^]^ Under controlled adipocyte‐beta cell co‐culturing, the IL6, TNF‐α, and insulin levels may be balanced to encourage normal glucose response and cytokine modulations, with insulin rescuing adiponectin secretion despite the elevated TNF‐α.^[^ ^56^ ^]^ However, when challenged with extended hyperglycemia, the dramatic uptick in TNF‐alpha, as seen in the results, can disrupt the physiological adipocyte‐beta cell crosstalk.^[^ ^56^ ^]^ Our newly developed flow‐enabled adipocyte‐beta cell crosstalk model‐on‐a‐chip can replicate these hyperinsulinemia and insulin resistance mechanisms observed in obesity, bringing us a step closer to understanding and designing therapeutic strategies to combat the disease.

## Conflict of Interest

The authors declare no conflict of interest.

## Supporting information



Supporting Information

## Data Availability

The data that support the findings of this study are available from the corresponding author upon reasonable request.
