# Supplementary material for: Modeling Adipokine and Insulin‐Mediated Crosstalk Between Adipocytes and Beta Cells Using Flow‐Enabled Microfluidics
Source: Small. 2025 Jul 31;21(35):2504686. doi: 10.1002/smll.202504686 (PMC12410909; doi:10.1002/smll.202504686)
Supplement: Supplementary file 1 — Supporting Information [file SMLL-21-2504686-s001.docx]

**Modeling Adipokine and Insulin-Mediated Crosstalk Between Adipocytes and Beta Cells Using Flow-Enabled Microfluidics**

*Mohamad Orabi^1,2^, Mehdi Sh. Yeganeh^1^, Tae-Hwa Chun^3,^*^♱^*, and Joe F. Lo^1,*^*

1: Department of Mechanical Engineering, University of Michigan, Dearborn, 4901

Evergreen Road, Dearborn MI-48128, United States of America

2: Department of Internal Medicine, University of Michigan, Ann Arbor, MI-48109, United States of America

3: Department of Internal Medicine, Division of Metabolism, Endocrinology & Diabetes, Biointerfaces Institute, University of Michigan, Ann Arbor, 22800 Plymouth Rd, Ann Arbor MI-48109, United States of America

*Corresponding author: Joe F. Lo (E-mail: jfjlo@umich.edu)

ORCiD: 0000-0001-5182-2563

♱ Co-corresponding author: Tae-Hwa Chun (E-mail: taehwa@med.umich.edu)

ORCiD: 0000-0001-9333-2589

**Working Principle of MicroTesla (µTesla) Pump**

The µTesla pump consists of a rotor made up of two parallel, smooth discs, each 10 mm in diameter and separated by a 1 mm gap (see Fig 1). The rotor was fabricated using fused filament fabrication (FFF) with ASA filament. After printing, small magnets were manually inserted into the base of the rotor. The entire assembly is enclosed within a cylindrical housing and positioned on top of a standard laboratory magnetic stirrer, which generates a rotating magnetic field. This magnetic field couples to the inserted magnets and transmits torque to the rotor, allowing it to spin without any direct mechanical contact.

This configuration leverages the boundary layer effect, a principle originally introduced by Nikola Tesla. As the discs rotate, viscous forces at the fluid–solid interface generate boundary layers that entrain adjacent fluid layers. The resulting velocity gradient across the disc surfaces produces tangential flow, causing the fluid to spiral outward. This radial acceleration induces a low-pressure zone at the center of the rotor, drawing fluid in through an axial inlet. The combined effect results in continuous suction and radial discharge, enabling bladeless, impeller-free pumping through viscous momentum transfer.


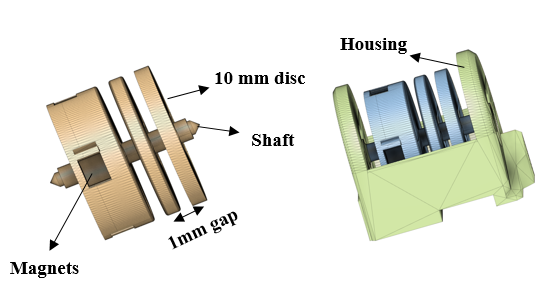


**Fig. S1.** Schematic of the rotor (left) and the pump assembly (right)


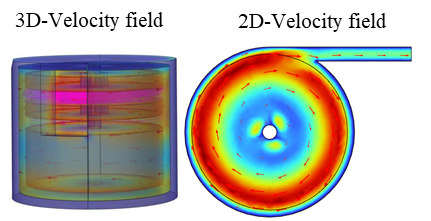


**Fig. S2.** 3D (left) and 2D (right) schematics of velocity spatial distribution exported from COMSOL Multiphysics


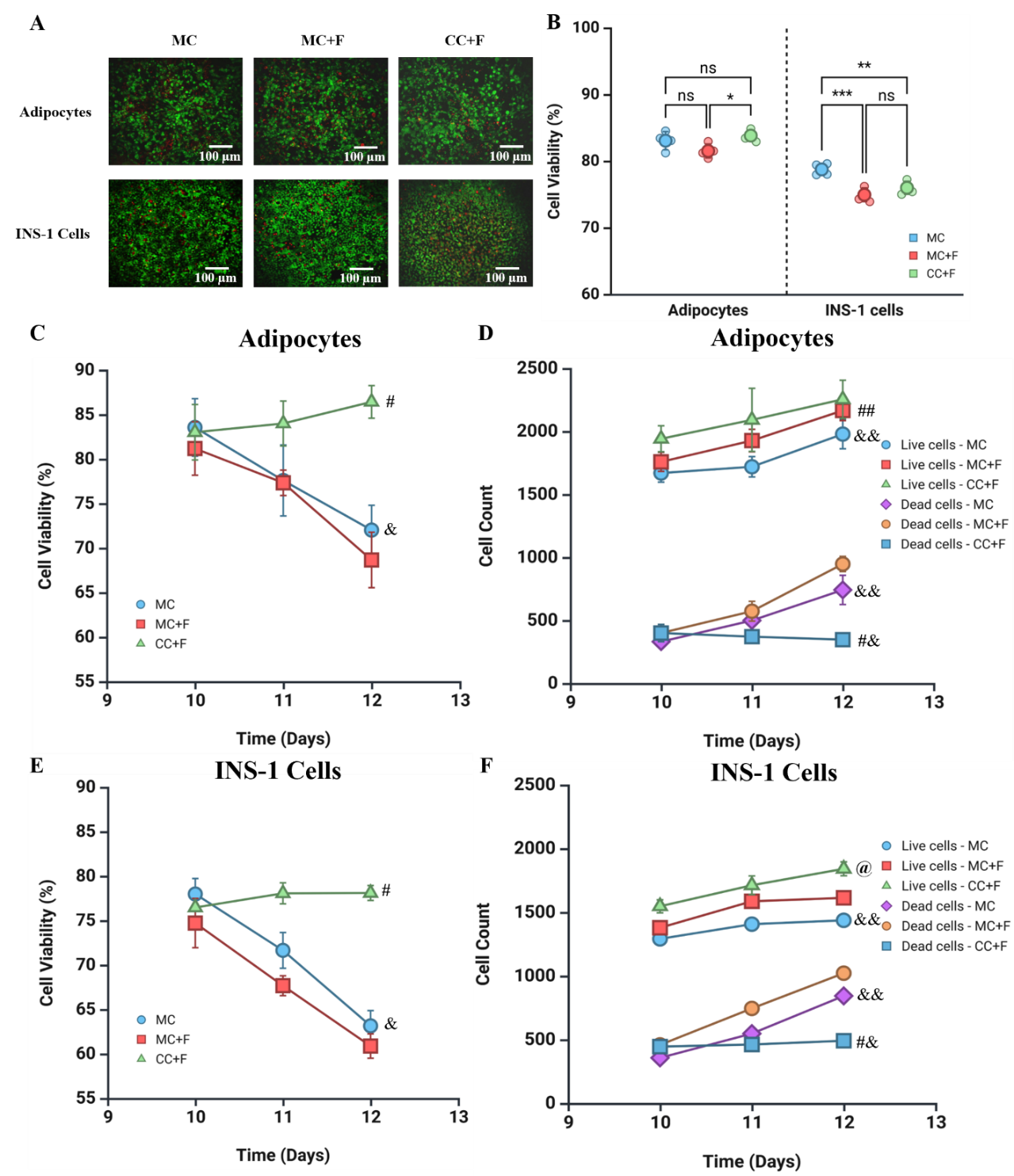


**Fig. S3. Impact of Flow on the Viability of Adipocytes and Beta Cells in Mono- and Co-Cultures**

(A) Fluorescence images of viability assays for monocultures under static conditions without flow (MC), with flow (MC+F), and adipocyte-beta cell co-culture with flow (CC+F). Adipocytes (top) and INS-1 rat beta cells (bottom). Images were taken using an Olympus IX75 microscope with a 10X lens and quantified using image J.

(B) The viability of adipocytes and beta cells at day 10.

(C) Viability improves over time for adipocytes co-cultured with INS-1 beta cells.

(D) Adipocyte viability results from decreased cell death and not proliferation. Cell count for live and dead adipocytes quantified with image J.

(E) INS-1 beta cell viability increased in co-culture with flow (CC+F).

(F) INS-1 live and dead cell counts.

# and ## are for day 12 comparing MC+F to CC+F for adipocytes and beta cells. & is for the same day comparing MC to MC+F for adipocytes and beta cells. && is for day 12 comparing live/dead cells MC to that of MC+F for both cell lines. #& is for day 12 comparing dead cells CC+F to that of MC+F. @ is for day 12 comparing CC+F to MC+F for INS-1 beta cells.

Data is presented as mean ± SEM, n=4. Statistical significance was assessed by using two-way ANOVA with Tukey multiple comparison tests. && and *P<0.05, @ and ***P<0.001, #, #&, and ****P<0.0001. & and ## means no significant difference. n stands for distinct experiments conducted in separate days.


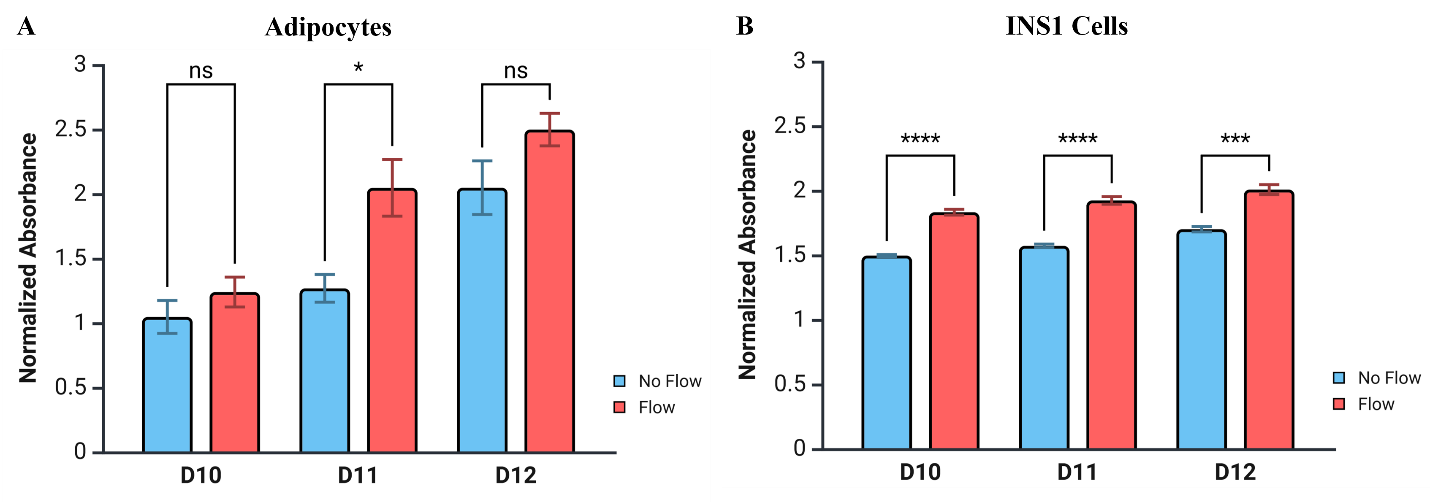


**Fig. S4. The normalized absorbance for adipocytes and INS1 cells.**

A) The normalized absorbance for adipocytes showed an increase with flow compared to no flow. For adipocytes without flow, exponential growth was recorded. However, with flow, the trend showed a linear increase over 3 days.

B) The normalized absorbance for INS1 cells showed a slight increase for flow compared to no flow. For the absorbance within the 3 days, a linear increase was shown with and without flow but with a lower slope compared to adipocytes with flow. Two-way ANOVA with Tukey multiple comparisons test was adopted to compare the data and conduct the analysis. Data is presented as means ± SEM, n=3. *P<0.05, ***P<0.001, ****P<0.0001. n stands for distinct experiments conducted in separate days.


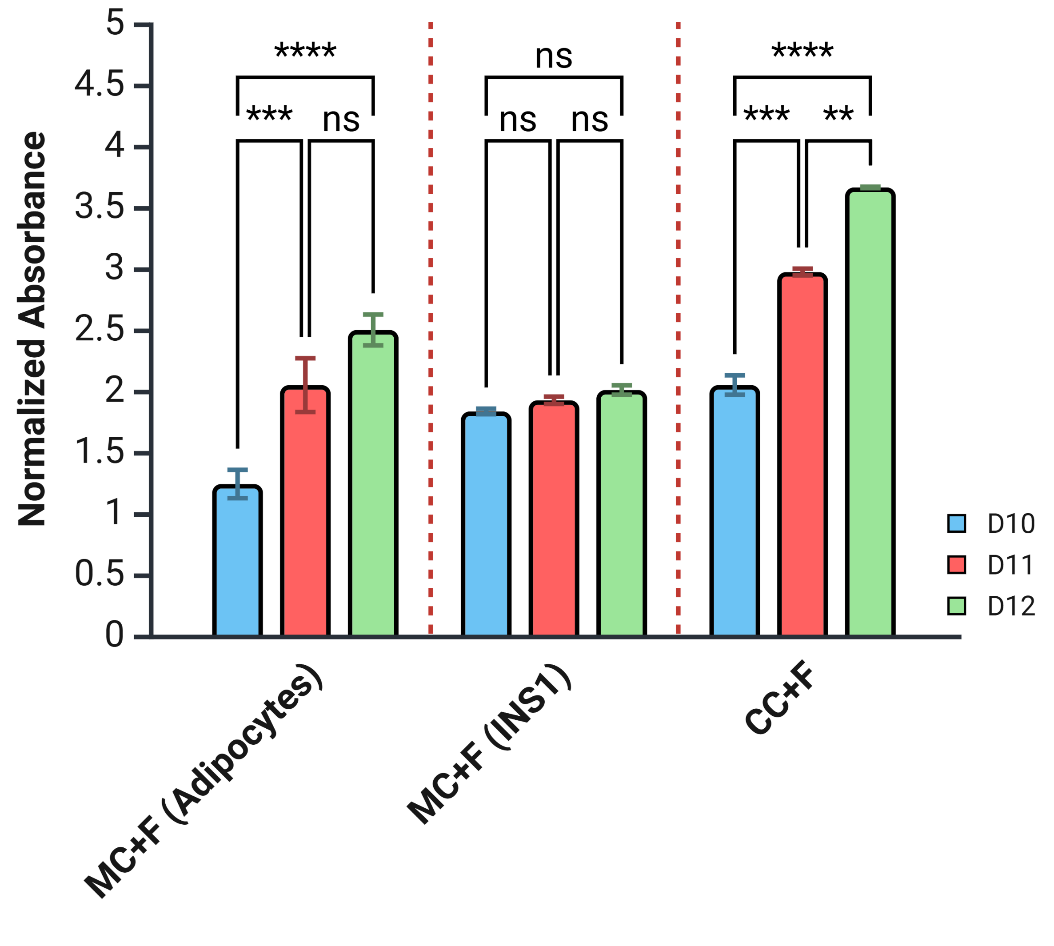


**Fig. S5. The normalized absorbance for adipocytes and INS1 cells with flow (MC+F) and the co-culture (CC+F) of both cell lines.**

The absorbance for INS1 and adipocytes was shown to be a linear increase but with a higher slope for adipocytes than INS1. The co-culture normalized absorbance has shown an increase too with a higher slope than adipocytes. Two-way ANOVA with Tukey multiple comparisons test was adopted to compare the data and conduct the analysis. Data is presented as means ± SEM, n=3. **P<0.01, ***P<0.001, ****P<0.0001. n stands for distinct experiments conducted in separate days.


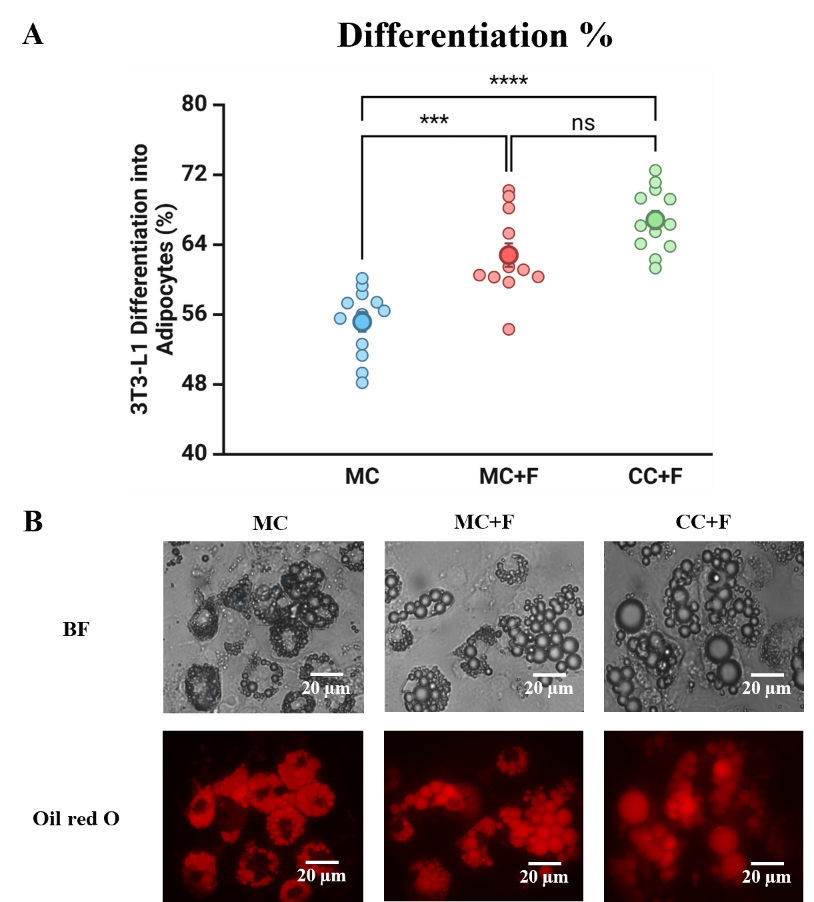


**Fig. S6*.* Increased adipocyte lipid accumulation in co-culture with flow**

(A) The percentage of differentiation was determined by dividing the count of lipid-containing cells (classified as particles in Image J based on the presence of lipid droplets) by the total number of cells (DAPI) in MC, MC+F, and CC+F conditions.

(B) Bright field (BF) and fluorescence images (Oil Red O) show lipid droplets inside individual adipocytes. The images were taken with an Olympus IX75 microscope with a 50X lens. Images were quantified using image J. Scale, 20 µm. One-way ANOVA followed by Tukey post hoc multiple comparisons was used for statistical analysis. Data is presented as mean ± SEM, n=3 in each experiment, experiments repeated at least 3 times. ***P<0.001, ****P<0.0001. n stands for distinct experiments conducted in separate days. The number of fields of view selected for analysis were 3 per each sample.


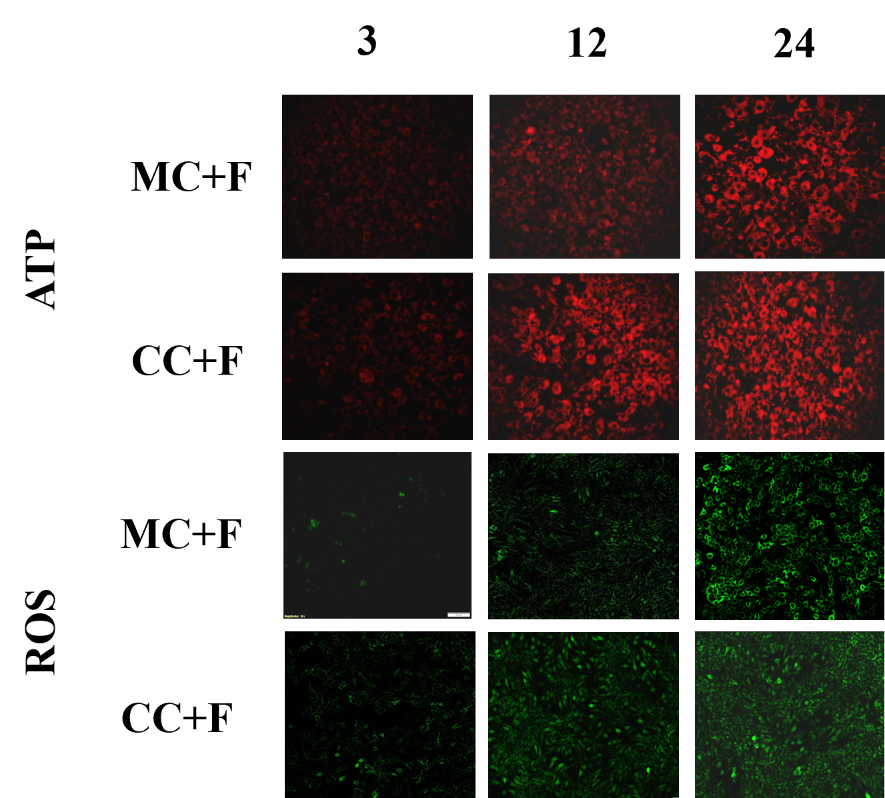


**Fig. S7.** Metabolic activity of adipocytes cultured for a total period of 24 hrs.


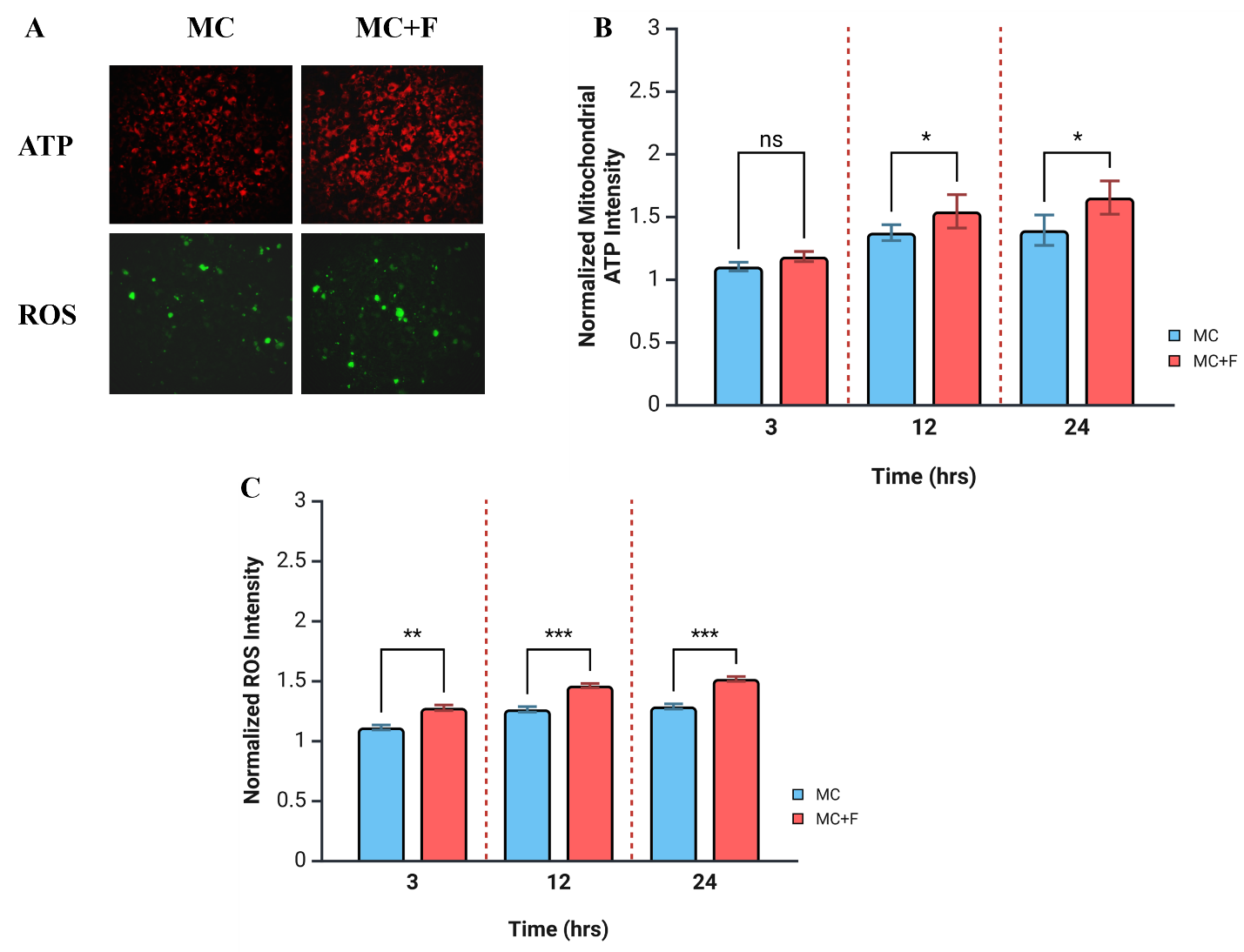


**Fig. S8. Morphological and metabolic enhancements of 3T3-L1 monocultures under no flow (MC)/flow (MC+F) conditions.**

**A)** Flow condition enhanced the ATP and ROS metabolic activity of 3T3-L1 cells.

**B)** The normalized mitochondrial ATP intensity for flow condition was higher than no flow with a bigger difference after 24 hrs.

**C)** The normalized ROS intensity has the same trend as ATP. The images were taken using an Olympus IX75 microscope with 10X magnification. Images were quantified using image J. Two-way ANOVA with Tukey multiple comparisons test was adopted to compare the data and conduct the analysis. Data is presented as means ± SEM, n=3. *P<0.05, **P<0.01, ***P<0.001. n stands for distinct experiments conducted in separate days.


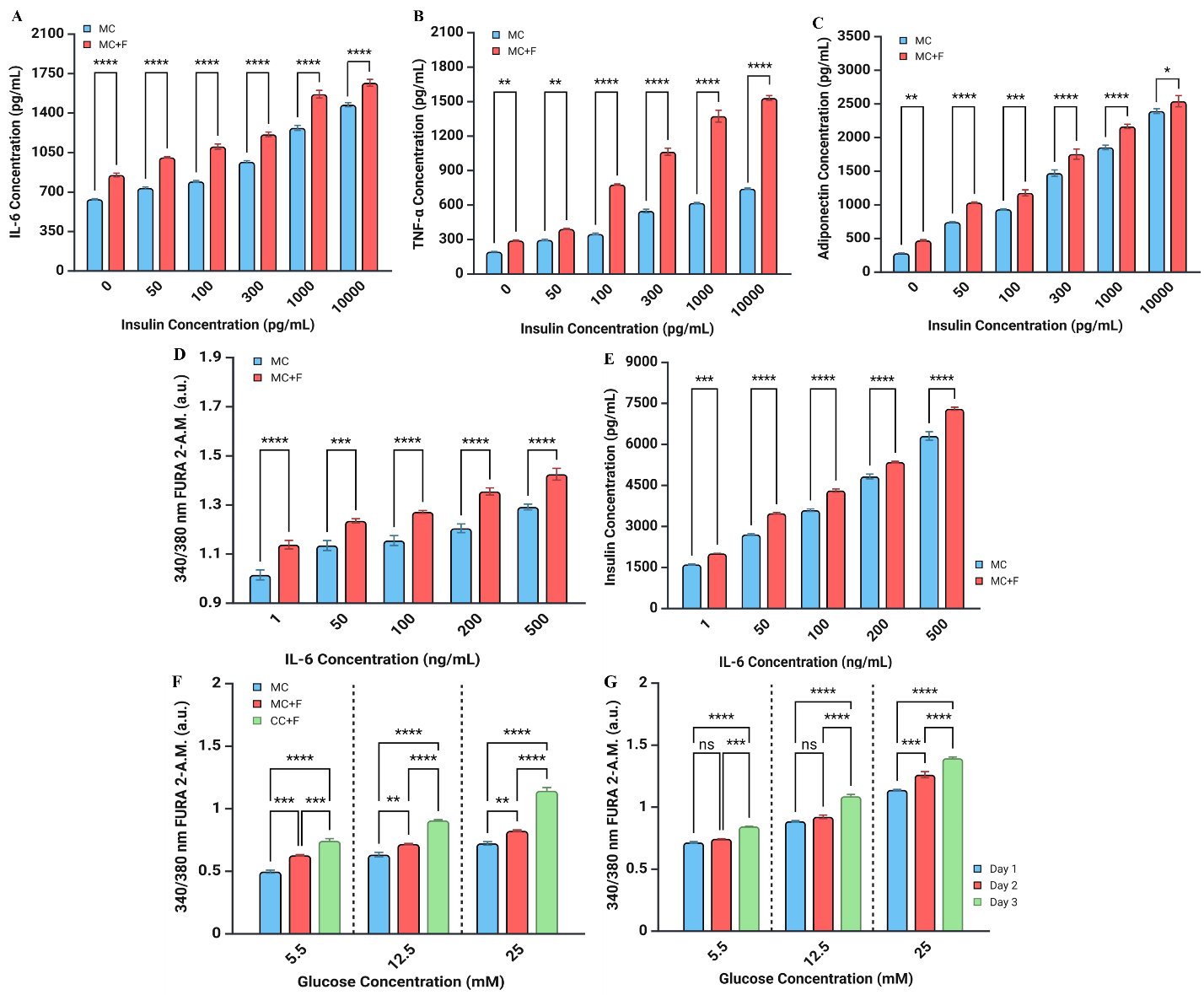


**Fig. S9. Flow augments the response of adipocytes and beta INS-1 cells to insulin and IL-6. Calcium responses for beta INS-1 cells.**

(A) IL-6 levels in response to increased concentration of insulin in adipocyte static monoculture without flow (MC) and with flow (MC+F). n=3

(B) TNF-α levels with increased insulin concentrations in adipocyte MC versus MC+F conditions. n=3

(C) Adiponectin levels with increased insulin concentrations in adipocyte MC and MC+F conditions. n=3.

(D) FURA (calcium flux) response to 12.5 mM glucose in the presence of increased concentrations of IL-6 in INS-1 MC and MC+F conditions. N=3.

(E) Insulin levels in INS-1 MC and MC+F conditions in the presence of increased concentrations of IL-6. n=3.

(F) INS1 calcium influx in response to glucose.

(G) INS-1 calcium influx was measured in adipocyte-beta cell CC+F incubated in the culture media with glucose 5.5 mM, 12.5 mM, and 25 mM for 30 mins.

The data were analyzed using a two-way ANOVA with Tukey's multiple comparison test. The two independent factors were culture conditions (MC vs. MC+F) and ligand concentrations (insulin and IL-6). The glucose concentration in the assay media used was 25 mM for adipocytes and 5.5 mM at baseline for INS-1 cells. Data are presented as means ± SEM, n=3. *P<0.05, **P<0.01, ***P<0.001, ****P<0.0001, ns: no significance. n stands for distinct experiments conducted in separate days.

## **IL6 Flow Dynamics in a Microfluidic Co-culture System**

The computational modeling of IL-6 flow dynamics within the microfluidic device demonstrates the transport of IL-6 from the adipocyte chamber to the beta cell chamber over time. At time 0 seconds, IL-6 is highly concentrated at the adipocyte chamber inlet, with no noticeable diffusion or transport. By 70 seconds, IL-6 begins to disperse, forming a diffusion front as flow-driven transport moves the molecules toward the beta cell chamber. At 150 seconds, IL-6 has significantly diffused, with a higher concentration observed within the beta cell chamber, indicating successful cytokine transport. The results highlight a combination of convection-driven transport and molecular diffusion, effectively facilitating cytokine exchange between adipocytes and beta cells. This microfluidic system efficiently models intercellular communication, making it a valuable tool for studying cytokine-mediated signaling in diabetes pathophysiology and assessing the impact of various flow rates and molecular gradients on cellular interactions.


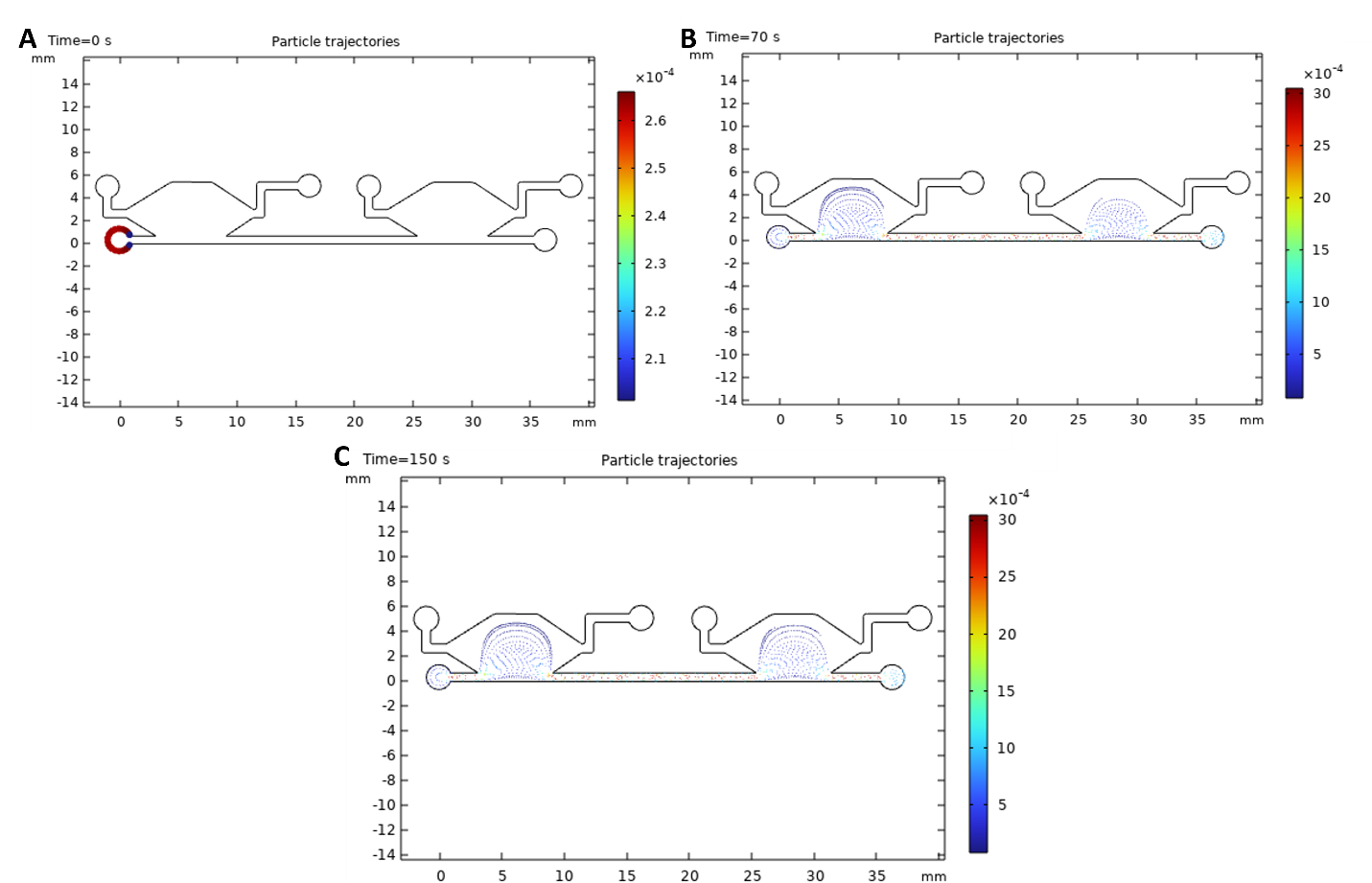


**Fig. S10. Computational modeling of IL6 flow dynamics within a microfluidic device**. It shows the transport of IL6 from the adipocyte chamber to the beta cell chamber over time (0s, 70 s, and 150 s).


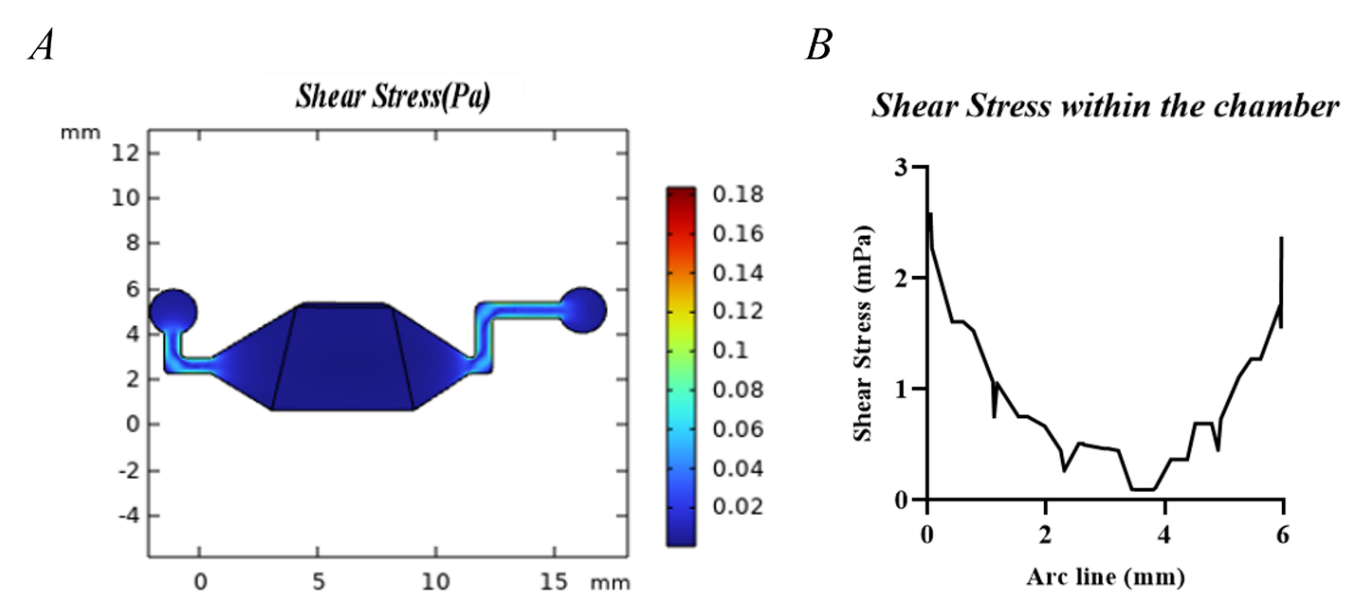


**Fig. S11.** **Computational analysis of shear stress distribution in the microfluidic chamber.** (**A**) Surface plot of shear stress (Pa) across the chamber geometry, demonstrating uniformly low stress levels in the central culture region. The chamber design promotes laminar flow and minimizes wall shear stress exposure. (**B**) Line profile of shear stress magnitude along the central axis of the chamber, highlighting a gradual decline toward the middle and confirming minimal shear stress within the main cell culture zone.
